# The Influence of Hydrogeomorphology on Food Webs in Riverine Landscapes

**DOI:** 10.1002/ece3.72299

**Published:** 2025-11-19

**Authors:** Martin C. Thoms, Michael D. Delong

**Affiliations:** ^1^ Riverine Landscapes Research Lab University of New England Armidale New South Wales Australia; ^2^ Large River Studies Center Winona State University Winona Minnesota USA

**Keywords:** complex adaptive systems, ecosystem function, environmental heterogeneity and variability, river ecosystems

## Abstract

A central tenet of river science is the interaction between flow and the physical habitat template—hydrogeomorphology—that governs biophysical structure and function. While studies have shown how this interplay shapes structural river ecosystem attributes, our knowledge of their influence on ecosystem function is limited. Using geomorphological, hydrological, and stable isotope ratio data for basal resources and primary and secondary consumers from 88 rivers, we test hypotheses on relationships between hydrogeomorphology and food chain length. A significant curvilinear relationship between the physical heterogeneity of a river reach and food chain length was found. Low flow variance was shown to have an additive influence on food chain length; longer food chain lengths occurred in reaches that experienced permanent flow but not the frequency of overbank floods. Ecosystem size had no effect on food chain length. The results of this study suggest reach‐scale hydrogeomorphology has a direct influence on ecological function—food chain length—in riverine landscapes. We suggest that the spatial heterogeneity of physical character is a primary driver of ecosystem function that provides a template upon which flow variability acts as a regulator of food chain length. Understanding biocomplexity, the interplay of spatial heterogeneity and temporal variability, is critical to predicting responses of riverine landscapes to natural and human‐derived disturbances.

## Graphical abstract

Reach scale hydrogeomorphology was shown to have a direct effect on food chain length. Low flow variance was shown to have an additive influence on food chain length; longer flood chain lengths occurred in reaches that experienced permanent flow but not the frequency of overbank floods. Ecosystem size had no effect on food chain length.

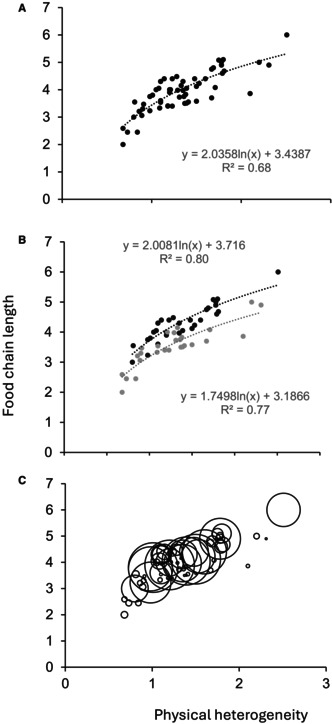

## 1 Introduction

Environmental heterogeneity and temporal variability are fundamental drivers of ecosystem structure and function (Levin 1992). Spatial heterogeneity provides a mosaic of habitats, and resources that support biodiversity (MacIntosh and Tonkin 2024; Yang et al. 2025), facilitate species coexistence (Stien et al. 2014), and enhances ecosystem resilience (Oliver et al. 2015). Temporal variability, operating across multiple scales shapes ecological processes by influencing species life histories, population dynamics, and community interactions (Chesson 2000; Ellner et al. 2016). Together, heterogeneity and variability underpin ecosystem complexity, mediate responses to disturbance, and promote the adaptive capacity of ecosystems in the face of environmental change (Folke et al. 2004; Oliver et al. 2015). Recognizing and incorporating this dynamic is central to ecological theory and for effective conservation and restoration strategies as well as sustainable resource management.

The biophysical character of riverine landscapes has been conceptualized to be the product of multiple abiotic and biotic interactions (cf. Townsend and Hildrew 1994; Thorp et al. 2008; Dodds et al. 2015; Allen et al. 2020). Geomorphology, particularly the spatial heterogeneity of physical character, provides a template upon which evolution acts to forge characteristic life history strategies (cf. Southwood 1977, 1988). Hydrology, through its temporal variance in flow, acts as a natural disturbance upon this template to regulate biogeochemical processes (Walker et al. 1995; Boltz et al. 2019). Thus, hydrogeomorphology—the interaction of geomorphology and hydrology—creates a dynamic mosaic of physical and geochemical properties that influence the type, abundance, arrangement, and persistence of biological assemblages and ecological functions across riverine landscapes. These interactions have been demonstrated in studies at multiple spatial scales. At the river network scale, river zones (i.e., lengths of river network between 5–50 km) with unique hydrogeomorphological properties (Thoms et al. 2017) have been shown to have different fish assemblages (Boys and Thoms 2006; Elgueta et al. 2019), which are correlated to larger‐scale catchment variables such as valley confinement, down‐valley slope, and the degree of connectivity between valley side and the river channel (e.g., Robbins and Pyron 2021; Shields et al. 2021). At smaller scales (i.e., 100–1000 m), river reaches are portrayed as a mosaic of physical units or patches, such as riffles, pools, and bars (e.g., Townsend and Hildrew 1994) for which species traits can be matched (Resh et al. 1994). Benthic macroinvertebrate diversity has been shown to be directly correlated to smaller scale hydromorphological instream habitat character and quality (Béjar et al. 2020; MacIntosh and Tonkin 2024). Biophysical complexity is an emergent property of riverine landscapes at multiple scales.

Studies examining the effect of hydrogeomorphology on ecosystem function in riverine landscapes are limited (Stien et al. 2014; Arga et al. 2024). This is despite numerous hypotheses stressing the importance of environmental heterogeneity and variability—complexity—as a key driver of ecosystem function (cf. Vander Zanden and Fetzer 2007; Takimoto and Post 2013; Scholl, Cross, and Guy 2023, Scholl, Cross, Guy, Dutton, and Junker 2023). Hydrogeomorphology has been suggested to be an important mechanism influencing food chain length (F_CL_) (cf. Hoeinghaus et al. 2008; Rossberg 2012). F_CL_ is a fundamental measure of the vertical organization of food webs, including the extent of food web complexity, energy transfers, and interactions within communities (Post 2002a, 2002b). Its central role as an integrator of community and ecosystem function is well known (Thompson et al. 2017). Despite the influence of physical character and flow on F_CL_ being addressed in food web studies (e.g., Delong et al. 2019), typically, physical or morphological heterogeneity and flow variability are investigated separately, with their interaction being an output rather than the direct focus of river ecosystem function investigations (Power 2006). For example, F_CL_ was shown to increase with the physical heterogeneity of the Kanawha River, USA (Thoms et al. 2017); whereas Warfe et al. (2013) suggested variations in hydrological connectivity create regional food webs that override the effect of ecosystem size. In another study, Sabo et al. (2010) determined F_CL_ to decrease with an increase in flow variability and concluded that flow variability, along with ecosystem size, drives F_CL_. Further, hydrogeomorphology variables are not commonly quantified in the study of F_CL_ in river ecosystems; rather, differences in environmental conditions between sites are assumed (e.g., Hoeinghaus et al. 2008; Thompson and Williams 2018). While previous studies provide insight into the influence of hydrogeomorphology on F_CL_, the key component of interactions between geomorphological heterogeneity and flow variability on ecosystem function within these complex systems is not directly incorporated in study designs. Despite reviews (e.g., Post 2002a, 2002b) and meta‐analyses (Vander Zanden and Fetzer 2007), debate on proximal drivers of ecosystem function (i.e., F_CL_) in rivers remains unanswered (Takimoto and Post 2013).

The findings of an interdisciplinary study that investigates the influence of hydrogeomorphology on river ecosystem function, and particularly F_CL_, are presented. Hypotheses proposed to explain F_CL_ variations in river ecosystems have focused on ecosystem size (Vander Zanden et al. 1999; McIntosh et al. 2024), dynamic stability (McHugh et al. 2010), productivity (Post 2002a, 2002b), and productive space (combining productivity and ecosystem size) (Schoener 1989). However, these hypotheses do not consider the potential effect of hydrogeomorphology as a primary driver of F_CL_ (Vander Zanden and Fetzer 2007; Takimoto and Post 2013). In reviewing influences on F_CL_, Post (2002a) suggested broadening the search for determinants to consider how interactive factors (i.e., physical heterogeneity and flow variability) may affect F_CL_. If riverine landscape complexity is a function of the interactive effect of hydrogeomorphology, we suggest physical heterogeneity (P_H_) will be the primary driver of F_CL_, with flow connectivity (a surrogate for hydrological variance (Thoms and Parsons 2003)) acting as a regulator. We hypothesize that F_CL_ will increase with P_H_, and increasing flow connectivity will have an additive influence on F_CL_. In addition, we hypothesize that ecosystem size will further enhance the combined effect of physical heterogeneity and flow variance—hydrogeomorphology.

## 2 The Study

### 2.1 Study Design

The objective of this study was to determine the relationship between hydrogeomorphology—the interaction of physical heterogeneity and hydrological variance—and food chain length, in riverine landscapes. Hydrogeomorphological and trophic data (stable isotope ratios of carbon (δ^13^C) and nitrogen (δ^15^N) for primary basal resources plus primary and secondary consumers) were obtained for 88 river reaches in Australia, Chile, and the United States of America from published and unpublished studies (Table 1). River reaches had a uniform morphology along a length of channel of approximately five kilometers i.e., there was no abrupt change in physical character along the reach.

**TABLE 1:** Information used in this study. Rivers, country of origin, the number of sites in each river, and the publication source for this information are provided.

River	Country	Number of reaches	Publication
Arkansas (Ar)	USA	2	Delong and Thoms (2016)
Barwon Darling (BD)	Australia	4	Thoms and Delong (2018)
Biobio (Bio)	Chile	4	Vagas et al. (2013) Thoms (unpublished)
Birrie (Bi)	Australia	4	Reid, Webb, and Thoms (2012)
Culgoa (Cul)	Australia	8	Reid, Webb, and Thoms (2012) Thoms (unpublished)
Illinois (Ill)	USA	4	De Boer et al. (2020)
Imperial (Im)	Chile	4	Thoms (unpublished)
Kanawha (Kan)	USA	4	Thoms et al. (2017)
Kansas (Ka)	USA	2	Delong and Thoms (2016)
Macintyre (Mac)	Australia	8	Reid, Delong, and Thoms (2012)
Missouri (Misso)	USA	2	Delong et al. (2011)
Narran (Na)	Australia	8	Reid, Delong, and Thoms (2012) Thoms (unpublished)
Ohio (Oh)	USA	2	Delong and Thoms (2016)
St Croix (SC)	USA	4	Delong et al. (unpublished)
Upper Mississippi (UMR)	USA	28	Delong and Thoms (2016) Delong et al. (unpublished)

### 2.2 Physical Character and Hydrological Variance

For each river reach, a set of morphological variables (Table 2) representing the size, shape, and efficiency of the bankfull channel was collected from remotely sensed data and field‐based surveys. The planform character of each reach (i.e., river channel sinuosity, meander wavelength, and shoreline complexity) was determined from remotely sensed images. Field‐based surveys consisted of a series of 25 bankfull cross‐sectional channel surveys undertaken at ~200 m intervals along each reach. Ecosystem size was calculated as the channel capacity of the river reach at bankfull stage (R_CC_; Table 2), where biological specimens were collected for the food web analyses. In addition, the physical heterogeneity (P_H_) of each river reach was determined according to the rank dissimilarity used to compute the comparative index of multivariate dispersion (IMD), as described by Warwick and Clarke (1993). IMDs were determined from an ordination of the morphological variables collected at the 25 sites along each reach. For this, the Gower association measure, a range‐standardized measure and recommended for nonbiological data (Belbin 1993), was used in the semi‐strong hybrid multidimensional scaling (SSH) ordinations. This multivariate measure of rank dissimilarity (i.e., IMD) was adopted as a measure of the spatial heterogeneity in physical character—physical heterogeneity (P_H_) for each river reach. This multivariate measure of variance has been used to describe spatial variance in reach‐scale hydraulic environments (Dyer and Thoms 2006; Thoms et al. 2006) and changes in river channel morphology within river networks (Thoms et al. 2017; Elgueta et al. 2020; Bajracharya et al. 2023).

**TABLE 2:** Physical variables measured at each river reach.

Variable		Description
Notation		
*Size*		
W	Channel width (m)	Width of bankfull channel
D	Channel mean depth (m)	Mean depth of bankfull channel
CC	Channel area (m^2^)	Cross‐sectional at bankfull level
Rcc	Reach capacity (m^3^)	The volume of the river at bankfull level
Wp	Wetted perimeter (m)	Length of river section at bankfull
*Shape*		
F	Width‐depth ratio (m)	A measure of channel shape related to sediment transport and boundary conditions
A*	Channel asymmetry 1 (m^2^)	A* = (Ar—Al)—CC, where Ar and Al are the areas to the right and left of the channel center line, and CC is the channel cross‐section area.
Az	Channel asymmetry 2 (m)	Az = 2*x* (*D*_max_–*D*) + CC, where *x* is the distance from the channel centerline and *D*_max_ is the maximum depth of the cross‐section.
Rir	Channel irregularity (d)	Ratio of the Wp of the cross section to the Wp of the same CC expressed as a semi‐circle.
SLcx	Shoreline complexity (d)	Ratio of the length of the river reach to the circumference of a circle with the same surface of the river reach. SLcx = *L*/2√*πA*, where *A* is the reach surface area.
*ρ*	River channel sinuosity (d)	Ratio of river channel reach length to the straight line valley length.
*λ*	River channel meander wavelength (m)	Distance between the apex meander along the down‐valley axis.
*P*_H_	Physical reach heterogeneity (d)	Spatial heterogeneity in the physical character of a reach. Full details of this measure are provided in the text.
*Efficienc*y		
Hr	Hydraulic radius (m)	Hr = CC + Wp is a measure of channel efficiency, which, for a given channel size, reaches a maximum for a semi‐circular channel
K	Conveyance factor (m)	*K* = CC^1.66^ Wp^−0.66^

Abbreviations: d, dimensionless; m, meters; m^2^, meters squared; m^3^, cubic meters.

Two measures of flow variance were calculated from long‐term daily flow records (*n* > 30 years) of the nearest gauging station. Based on the degree of hydrological connection at low flow (HC), river reaches were classified as either permanently connected (P) or intermittently connected (I) (i.e., periods of no flow), the latter having an average annual frequency of flow disconnection of more than 30 days along the river reach. High flow connectivity (F) with the adjacent floodplain was determined as the annual exceedance of bankfull discharge for each reach. These flow measures have been successfully applied in flow ecology studies (cf. Gordon et al. 2004).

### 2.3 Food Chain Length

Data for food chain length calculation consisted of carbon (δ^13^C) and nitrogen (δ^15^N) stable isotope ratios of samples for primary basal resources plus primary (e.g., invertebrates) and secondary (e.g., fish) consumers. These data allowed the calculation of trophic position and food chain length for each river reach. Trophic position, a continuous measure of the location of consumers on a food chain, was calculated following the method of Post (2002a, 2002b) using the equation: trophic position = *λ* + (δ^15^N_sc_–[δ^15^N_base1_ *α* + δ^15^N_base2_ (1–α)])/Δ_*n*_: where *λ* = trophic position of primary consumers (= 2); δ^15^N_SC_ = the δ^15^N of the fish; and Δ_*n*_ = fractionation of δ^15^N from prey to consumer. The parameter α was determined by *α* = (δ^13^C_SC_—δ^13^C_base2_)/(δ^13^C_base1_—δ^13^C_base2_) as recommended by Post (2002b). Where isotope ratios were not available for basal resources along a reach, benthic invertebrates known to feed primarily on pelagic basal sources or benthic basal sources were used as surrogates for basal sources (Post 2002b). The mean trophic position of five functional fish feeding guilds (i.e., trophic guilds) identified in each river reach was calculated, with the functional feeding guild having the highest mean trophic position assigned to represent food chain length. Functional guilds are regularly used in diversity studies of freshwater fishes (e.g., Welcome et al. 2006; Cowx and Portocarrero Aya 2011). For this study, fishes were assigned to five different functional feeding guilds—herbivore, invertivore, omnivore, piscivore, and planktivore—according to the criteria of Poff and Allan (1995) and O'Hara et al. (2007).

### 2.4 Statistics

The large dataset generated for this study (88 reaches by 16 isotope ratios—i.e., 2 basal resources, 1 primary consumer, and 5 functional fish feeding guilds—by 15 hydrogeomorphological variables) was analyzed using a variety of multivariate statistical techniques to initially identify groups of river reaches with similar food web character, and which geomorphic variables may contribute to variance in food chain length, among river reaches. The isotope data were classified using the flexible unweighted pair‐group method with arithmetic averages (UPGMA) fusion strategy, as recommended by Belbin and McDonald (1993), based on the 16 isotope variables. Groups of reaches with similar isotope character were selected from the dendrogram representation of the cluster analysis, whereby the least number of groups with the maximum similarity was chosen. This step required the identification of an inflection point in the relationship between the number of groups in the classification and their corresponding similarity value. Dendrograms rarely display a linear increase in the number of groups; more commonly, the number of groups increases exponentially with increasing similarity (Thoms et al. 2017). This approach has been successfully applied in different environmental applications, including sediment textural analysis (Forrest and Clark 1989), catchment hydrology (Thoms and Parsons 2003), and stream network morphology (Thoms et al. 2018). Next, a SIMilarity PERcentage analysis (SIMPER; Clarke 1993) was undertaken to determine which geomorphological and flow variables contributed to the within‐group similarity, as identified in the cluster analysis.

Finally, least‐squares regression was used to examine the relationship of ecosystem size (river reach capacity—R_CC_), physical heterogeneity (P_H_), and flow variance (HC and F) to F_CL_. Analysis of variance (ANOVA) was used to test the significance of the slope generated by each least squares regression. Analysis of covariance (ANCOVA) was applied to test for differences in slope between those generated by least‐squares regression, and an *F*‐test determined if there were differences in exponents (Snedecor and Cochran 1991). A bubble plot was generated to examine further linkages between ecosystem size (R_cc_), physical heterogeneity (P_H_), and food chain length (F_CL_).

## 3 Results

Four trophic groups emerged from the multivariate statistical classification of the 88 reaches based on the stable isotope ratios of basal resources, primary consumers, and functional fish feeding guilds (Table 3). These four groups explain 82.3% of the similarity between the river reaches used in this study. No discernible pattern of individual reaches was found among the four groups. However, Group 1 comprises reaches from the USA, Groups 2 and 3 have a combination of reaches from Australia, Chile, and the USA, while Group 4 has reaches from Chile and the USA only (Table 3). For individual variables, despite differences in the range of δ^13^C and δ^15^N ratios among the four groups (Table 3), these were not significant (ANOVA: *F* = 7.05, *p* = 0.35). Overall, food chain lengths (F_CL_) ranged from 2 to 6 (Table 3) for the 88 reaches and were within ranges reported elsewhere in the literature (typically between 3 and 5 trophic levels, Pimm 2002). F_CL_ differs significantly, albeit at a lower significance level (ANOVA: *F* = 28.1, *p* < 0.1), between some of the four groups (Table 3), i.e., Group 1 differs from Groups 2 and 3, and Group 2 differs from Groups 3 and 4.

**TABLE 3:** River reach trophic groups and associated hydrogeomorphological variables.

		Trophic group			
		1	2	3	4
River reach composition		Ar1–2 Ill1–4 Ka2–4 Kan2 Misso2 UMR5–10	BD1–4 Bi1–3 Cu1–6 Mac1–8 Na1–8 UMR20–28	Bi4 Cul7 Cul 8 Misso3–4 Oh1 Oh2 UMR1–4 UMR11–19	Bio1–4 Im1–4 Misso1 Ka1 Ka 1 SC1–4
Bankfull dimensions		200–1625 m	83–1578 m	45–1641 m	121–1719 m
Flow character	Low flow (HC)	P, I	P, I	P, I	P, I
	Flood (F—ARI)	2–3.6	2–5.8	2–6.5	2–3.8
δ^13^C		−19.9 to –26.8	−18.1 to –29	−20 to –28.1	−14.8 to –32
^15^N		13.4–18.5	5.9–18.2	8.9–18.1	13–16
Food chain length (F_CL_)		2, 3* 3.6–6	3, 4* 2.1–4.8	2–6.2	3–5.8
Hydro—geomorphological variables*		P_H_ Rir SLcx K HC	P_H_ Rir K HC F	P_H_ R_cc_ Rir K F	P_H_ HC

*Note:* Rivers: Ar, Arkansas; BD, Barwon Darling; Bi, Birrie; Bio, Biobio; Cul, Culgoa; Ill, Illinois; Im, Imperial; Ka, Kansas; Kan, Kanawha; Mac, Macintyre; Misso, Missouri; Na, Narran; Oh, Ohio; SC, St Croix; UMR, Upper Mississippi (cf. Table 1). Bankfull dimensions: the range in bankfull widths for the reaches used in the study. Flow character: Low flow connectivity (HC)—(P) Permanently connected at low flow; (I) Intermittently connected at low flow. Flood (F—ARI) —the Average Recurrence Interval of over bank flows. Food chain length (F_CL_): * significant difference *p* < 0.1, ** significant difference *p* < 0.05. The bold numbers indicate which groups are significantly different to each other. Hydrogeomorphology variables: F‐ width to depth ratio of bankfull cross section; HC, hydrological connectivity at low flow; K, conveyance factor; P_H_, physical reach heterogeneity; Rcc, River reach capacity at bankfull (cf. Table 2). Rir, channel irregularity; SLCx, Shoreline complexity.

The SIMPER analysis showed seven hydrogeomorphological variables, representative of the size, shape, and efficiency of the bankfull channel morphology, river channel planform, and flow variance at high and low flows, explained a high degree (83%, 85%, 81%, and 86% for Groups 1, 2, 3, and 4, respectively) of within‐group similarity (Table 3). Physical reach heterogeneity (*P*_H_) was the only variable common to all four groups, while the size of the reach, calculated via river reach capacity (R_CC_), was associated with only one group (Group 3). Further, flow variance at low (HC) and high flow (*F*) was associated with three and two of the four groups, respectively.

**FIGURE 1:**
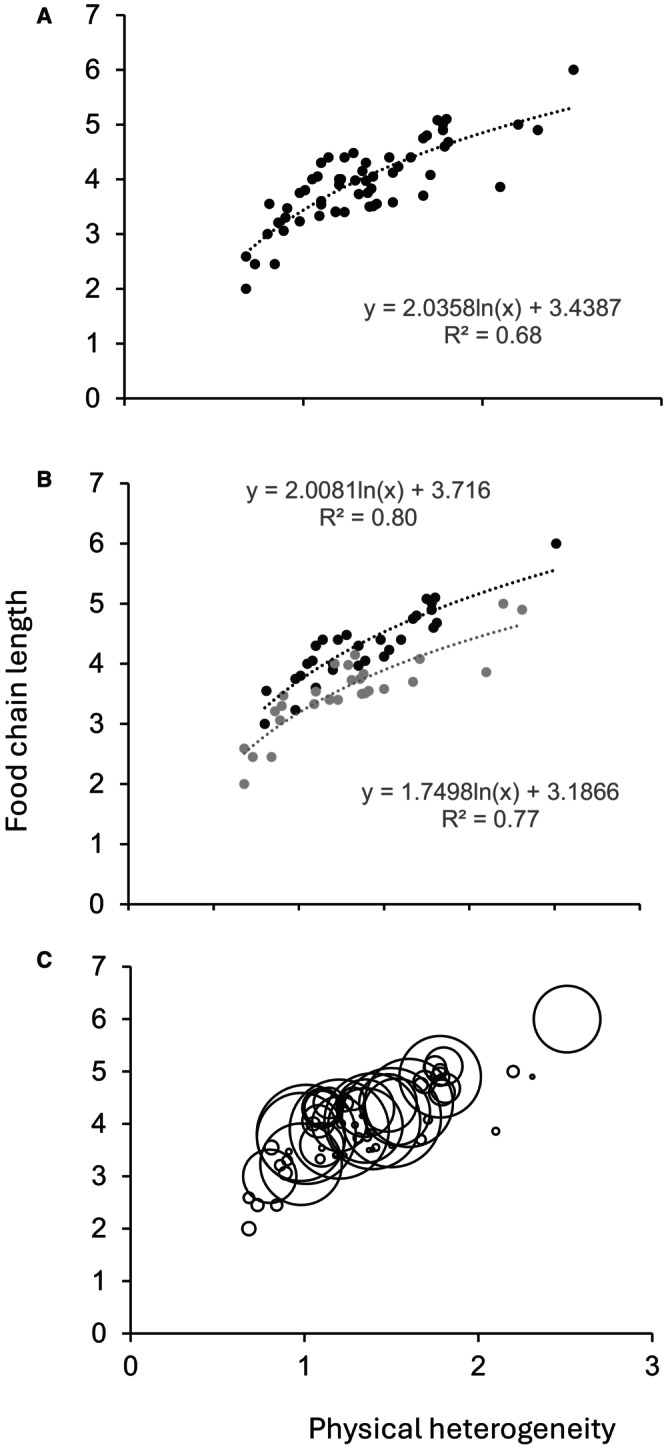
Hydrogeomorphology and food chain length for the 88 sites examined. Relationships for: (A) Physical heterogeneity and food chain length. (B) Physical heterogeneity, flow variability, and food chain length. (C) Physical heterogeneity, ecosystem size, and food chain length, with the size of the bubble equating to the ecosystem size. Solid dots are the permanently connected sites (P), and the gray dots are the intermittently connected sites (I).

A significant curvilinear relationship (ANOVA: *p* < 0.05; *r* ^2^ = 0.687) exists between physical heterogeneity (P_H_) and F_CL_ (Figure 1a). Thus, relative increases in F_CL_ associated with increasing P_H_ decline. For example, a 0.25 increase in P_H_ between 1–1.25, 1.7–2.15, 2.8–3.05, and 3.7–3.95 is associated with corresponding increases of 0.19, 0.11, 0.07, and 0.05 in F_CL_, respectively. Beyond a P_H_ of 4, increases in F_CL_ are minor, i.e., < 0.05. Similar significant curvilinear relationships also exist for both permanently (P—ANOVA: *p* < 0.05; *r* ^2^ = 0.802) and intermittently connected river reaches (I—ANOVA: *p* < 0.05; *r* ^2^ = 0.775) (cf. Figure 1b). These relationships are significantly different from one another in terms of the exponent (*F*‐Test: *p* < 0.05) but not slope (ANCOVA: *p =* 0.285) (Figure 1b). Food chain lengths are consistently longer in permanently (P) connected river reaches compared to intermittently (I) connected river reaches by an average of 0.53 trophic levels, i.e., at the same level of P_H_, F_CL_ is longer in the permanently (P) connected river reaches compared to those in intermittently (I) connected river reaches. No significant difference in F_CL_ was found among reaches of different degrees of flood frequency (i.e., F—ARI = 2–3, F—ARI = 3.1–4, F—ARI > 4.1; ANOVA: *p >* 0.451).

No significant relationship exists between F_CL_ and ecosystem size (R_CC_) for the combined data (ANCOVAR: *p* = 0.417), the permanently connected river reaches (p—ANCOVAR: *p* = 0.389), or the intermittently connected river reaches (I—ANCOVAR: *F* = 12.89, *p* = 0.721) (Figure 1c). These variable relationships (cf. Figure 1) dictate that we accept the Null hypothesis and suggest that P_H_ is a primary driver of F_CL_, and flow variability, particularly at low flow, is a regulator in riverine landscapes. Further, we reject the Null hypothesis that river reach size has a direct influence on F_CL_.

## 4 Discussion

Our results show hydrogeomorphology—the interaction between spatial heterogeneity in physical character (P_H_) and flow variability through its role of low flow connectivity (HC)—to be a primary influence on food webs, specifically F_CL_ in the riverine landscapes studied (Table 3; Figure 1). This finding contributes to our understanding of the interactive effect of geomorphology and hydrology as a driver of ecosystem function in rivers. Studies of relationships between the biophysical template and ecosystem function have focused on ecosystem size (e.g., MacIntosh et al. 2024) and/or habitat diversity (e.g., Scholl, Cross, and Guy 2023). Ecosystem size influences are integral to the understanding of biophysical dynamics in rivers. Established river models (e.g., River Continuum Concept) identify the effects of size‐related drivers with distance downstream with corresponding increases in area, the number of niches, and resource availability (Vannote et al. 1980). Positive relationships between total resource availability and F_CL_ have been shown for many aquatic ecosystems (cf. Post 2002a, 2002b). Further, the Flood Pulse Concept of Junk et al. (1989) suggests changes in ecosystem function are linked to flood‐mediated lateral expansion and contraction of riverine landscapes. A recent meta‐analysis of ecosystem effects of inundation showed that the larger the flood (lateral expansion), the larger the transfer of carbon to the river channel from floodplains (McInerney et al. 2023). Floodplain and riparian allochthonous pulses can sustain in‐channel secondary production for significant periods (Robertson et al. 2016). In addition, secondary production has been shown to differ among in‐channel habitats. A study by Scholl, Cross, and Guy (2023) showed patterns of river‐bed sediment texture at the local scale (< 100 m) govern invertebrate diversity, which propagated to drive positive relationships between habitat type and secondary production in the Yellowstone and Missouri Rivers. Further, changes in the spatial pattern of secondary production along the Yellowstone and Missouri Rivers study reaches were suggested to relate to the presence of ‘rare or patchy’ habitats and the magnitude of difference in sediment texture between a habitat type and the background habitat matrix rather than reach habitat diversity (Scholl, Cross, and Guy 2023). Although in a later study, Scholl, Cross, Guy, Dutton, and Junker (2023) showed increases in the heterogeneity of the river‐bed sediment environment led to weaker species interaction strengths, promoting stabilizing food‐web architectures in a single reach of the Missouri River.

Ecosystem theory suggests that size and environmental heterogeneity are two fundamental determinants of ecosystem character (Fath 2019). Larger ecosystems or those with a greater spatial heterogeneity promote diversity via increases in the total number of niches and/or available resources. Direct relationships between ecosystem size and F_CL_ have been shown for river ecosystems (cf. Post 2002a, 2002b; Power 2006). Our study shows a direct effect of the physical heterogeneity (P_H_) of river reaches on F_CL_ but no effect of size (Figure 1a,c). We suggest that increasing P_H_ will influence the number of niches per unit area of an ecosystem (Scown et al. 2015), with a corresponding increase in available food resources and species richness, thus F_CL_. The measure of heterogeneity (P_H_) in this study depicts spatial variance in physical character, in multidimensional space, and not just the presence, absence or amount of specific physical attributes as presented in previous studies (e.g., Scholl, Cross, and Guy 2023; Scholl, Cross, Guy, Dutton, and Junker 2023). However, the curvilinear relationship found in our study suggests the effect of P_H_ on F_CL_ ultimately reaches a threshold of influence beyond which biotic factors may become relatively more important. This may reflect the unimodal nature of the flow‐related area–heterogeneity relationship (cf. Allouche et al. 2012) in the river systems used for this study. Moreover, we acknowledge there are many ways to calculate ecosystem size from a physical and ecological perspective. The bankfull volume of a river reach, as used in this study, is one approach. Given river ecosystems are dynamic over time, and this is driven in part by fluctuations in flow, the calculation of ecosystem size at different discharges, including over bank flows, and their association with F_CL_ warrants further study.

Potential interactions between P_H_, ecosystem size, and F_CL_ in rivers must be acknowledged. Although we did not attempt to determine these interactions directly, our results warrant further investigation to define their extent and implications on food webs and food chain length in rivers. The *P*_H_ variable, as used in this study, calculates only one component of heterogeneity. There are two components to the spatial heterogeneity of river reaches. The first component, as calculated in this study, relates to the presence/absence, abundance, and diversity of geomorphic features present at a reach scale (lengths of river channel up to 5 kms). This influences the number and range of potential habitats and interactions between habitats, both of which contribute to heterogeneity (Levin 1998; Phillips 2003). The second component is concerned with the spatial organization or arrangement of geomorphic features or physical habitat present within a river reach. Spatial organization affects local interactions and feedback between physical features of any landscape, as well as the flux of matter and energy throughout the ecosystems present (Wiens 2002). The arrangement of large sediment clasts (boulders) and the degree of sediment sorting or mixing with finer sediment, on the riverbed can provide a mosaic of habitats (Ashmore and Rennie 2012) that may influence ecosystem structure and function at smaller scales (Harris et al. 2024). Further advances in understanding the interactive effect of geomorphology and hydrology on relationships between P_H_, ecosystem size, and F_CL_ must incorporate both components of spatial heterogeneity at multiple scales.

Environmental variability, whether it be rainfall or temperature, is an acknowledged regulator of the structure and function of ecosystems (Fath 2019). This occurs in riverine landscapes primarily via changes in the magnitude, frequency, duration, timing, and rate of change in flow (Richter et al. 2003). Scale‐dependent physical and biogeochemical relationships with flow have been studied for decades (cf. Schumm 1977; Walker et al. 1995), but relatively little is known of how specific components of the flow regime, and especially the magnitude, frequency, duration, timing and rate of change in flow connections at a range of temporal scales, affect the structure and functioning of food webs in river ecosystems. The flow regime has multiple roles in river ecosystems. Floods are not only associated with changes in ecosystem size but also are responsible for the redistribution of carbon, nutrients, organisms, propagules, and sediment in the longitudinal, lateral, and vertical dimensions of riverine landscapes (Humphries et al. 2024). In doing so, floods alter available resources that affect the structure and interactions of components of food webs. Floods are also recognized as a natural disturbance in rivers, whereby, on exceeding the threshold of motion, the movement of river‐bed sediment results in a ‘resetting’ of the physical environment with changes to basal resources, species composition, and trophic interactions. The debate about whether F_CL_ shortens or lengthens as a result of flood disturbance remains. Periods of hydrological disconnection may also alter trophic structure in river ecosystems. Ecological theory and some empirical studies suggest that smaller ecosystems should support shorter food chains because of reduced species diversity, habitat availability, and habitat heterogeneity, which in turn limit niche differentiation, feeding specialization, and enhanced levels of omnivory (Post 2002a, 2002b). However, the study by Reid, Delong, and Thoms (2012), which showed F_CL_ to be longer in low connectivity off‐channel ‘billabongs’ (permanent off channel or floodplain waterbody) compared to high connectivity billabongs, highlighted the compounding and complex effect of changing basal resources and trophic level interactions related to varying hydrological connections. Differences in the response of F_CL_ to flow variance have been hypothesized to relate to the type of basal resources present and their influence on inefficiencies in energy transfer through the food chain. Flow variability regulates access to available resources and exposure to biotic interactions across the riverine landscape (Sabo et al. 2010). Nonetheless, these influences may be mediated by the spatial heterogeneity in biophysical character (the biophysical template) of the river.

Biotic interactions have a significant effect on F_CL_ in river ecosystems, especially in more hydrologically stable environments (Hoeinghaus et al. 2008). Periodic hydrological disconnection was shown to diminish/eliminate access to basal food sources in the intermittently connected sites of the Upper Mississippi River by Delong et al. (2019). Moreover, increased duration of hydrological disconnections in the Upper Mississippi River resulted in a reduced potential for biotic interactions, such as predator–prey interactions, competition, and the effects of invasive species. We show F_CL_ to be longer in permanently connected reaches (P) at low flow, most likely from the greater availability and access to resources compared to those more intermittently connected river reaches (I) (Figure 1b). This may highlight the indirect effect of flow connectivity on ecosystem size.

Studies have successfully identified relationships between abiotic and biotic drivers of F_CL_ in river ecosystems (Power et al. 1996; Post 2002a, 2002b; McHugh et al. 2010). Despite this, it is apparent from the breadth of drivers tested there is no general model identifying a critical driver or set of influences on F_CL_. We agree with other researchers (Hoeinghaus et al. 2008; Post and Takimoto 2007; Sabo et al. 2010) that the search for single driver effects on F_CL_ is fruitless, and a shift towards examining interactive effects is more rewarding. Our study highlights the strong effect of P_H_ on F_CL_ and suggests the biophysical template to be the primary influence of ecosystem function, with flow variance acting as a regulator upon this template in riverine landscapes. Hydrogeomorphology serves as a framework in which to consider the interactive effect of heterogeneity and variability—complexity—on F_CL_ in riverine landscapes.

**FIGURE 2:**
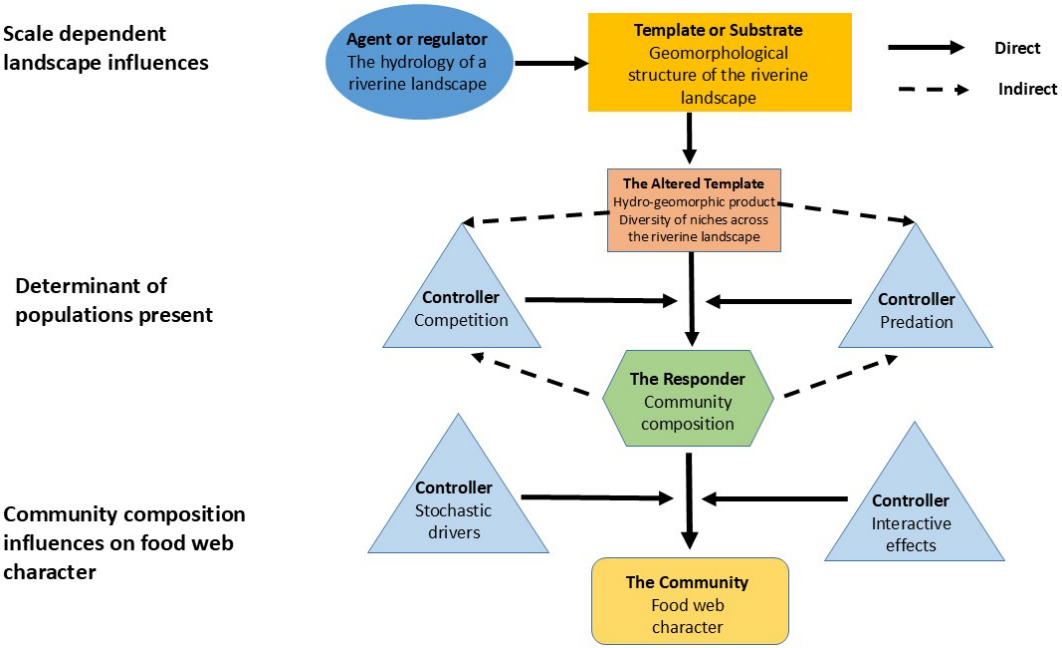
A conceptual framework for describing process interactions determining food chain length in riverine landscapes. The flow‐chain has four basic components: the abiotic or biotic agent or regulator of change, or driver; the template or substrate upon which the driver acts; controllers of the driver or agent of change; and an entity or process that responds to the driver or agent of change. Responders can be sets of processes, organisms, or parts of the physical environment.

Concepts of complexity go beyond the assumption that increasing ecosystem size begets more niches and available resources within river ecosystems. In riverine landscapes, ‘biocomplexity’ is the product of multiple interacting abiotic and biotic factors, and this can be represented via a conceptual framework (cf. Picket et al. 2007; Figure 2). Conceptual frameworks are a tool that can be used to order phenomena, reveal important patterns and processes at appropriate scales in environmental systems, structure working hypotheses and have been employed in understanding interactions between hydrology, geomorphology, and ecology at multiple scales (cf. Dollar et al. 2007). The conceptual framework provided illustrates both how riverine landscape heterogeneity can be generated and how ecosystem function responds to this heterogeneity (Figure 2). We suggest that the flow regime is the primary regulator of change that acts upon the physical template of the riverine landscape. The product of this interaction is the hydrogeomorphic landscape—the niche landscape. Niches within the riverine landscape are dynamic in time and space because of the regulating effect of hydrological variance. Aquatic communities are responders to this dynamic hydrogeomorphic landscape in this flow chain model. Controllers affect the action of the regulator of change on the transition from the hydrogeomorphic landscape to the response of aquatic communities. Predation, competition, interactive effects and stochastic events are key controllers influencing community composition within riverine landscapes. These controllers interact via a series of feedback loops between the hydrogeomorphic landscape and life history traits to modify the response of the aquatic community across the riverine niche landscape. This cascading chain of interactions is suitable for multiple scale settings, with both the regulator and the template able to be delineated at different scales. Our study contributes to the importance of hydrogeomorphology in understanding food web organization of freshwater ecosystems globally, as seen in the influence of physical heterogeneity interacting with hydrological variability on F_CL_.

Human domination over riverine landscapes has resulted in changes to the spatial arrangement and composition of physical character and associated flow regimes, among other things (Thoms and Fuller 2024). These changes have the potential to disrupt and reshape the architecture of species interactions that define patterns of energy flux, interaction strengths, and community stability measured by F_CL_. Accordingly, efforts to restore, rehabilitate, and repair rivers in the ‘Anthropocene’ have increased significantly in the last several decades. Adaptive management of the magnitude, frequency, and duration of river flows—Environmental flows—is a common approach for supporting freshwater‐dependent ecosystems and improving river health and biodiversity (Richter et al. 2003, Horne et al. 2017). This strategy is based on the principle that the flow regime is the ‘master variable’ that drives the function of river ecosystems. It has been a dominant paradigm of river science for more than 25 years (Gilvear et al. 2016). There are numerous empirical and conceptual studies, from various geographic and climatic regions, demonstrating the role of the flow regime in driving form and function in river ecosystems, at multiple scales (cf. Yarnell et al. 2024). Given the interactive effect of P_H_ and flow variance on ecosystem function, as shown in this study, it is incorrect to assume that flow is the only management action for restoring river ecosystems. Greater emphasis on hydrogeomorphology and its spatial or temporal character in river ecosystems would promote more effective river restoration.

## Author Contributions

**Martin C. Thoms:** conceptualization (lead), data curation (equal), formal analysis (lead), funding acquisition (equal), investigation (lead), methodology (lead), project administration (equal), resources (equal), software (lead), supervision (equal), validation (equal), visualization (lead), writing – original draft (lead), writing – review and editing (lead). **Michael D. Delong:** formal analysis (equal), funding acquisition (equal).

## Conflicts of Interest

The authors declare no conflicts of interest.

## Data Availability

All data is available in the main text of the manuscript from the University of England—www.une.edu.au. Data are held within the University of New England, Australia, data repository: https://doi.org/10.25952/b4zn‐0k53.
